# A preliminary survey of the cellular responses of the black fungus *Cryomyces antarcticus* to long and short‐term dehydration

**DOI:** 10.1111/1758-2229.13309

**Published:** 2024-07-29

**Authors:** Cassaro Alessia, D' Alò Federica, Pacelli Claudia, Cavalazzi Barbara, Zucconi Laura, Onofri Silvano

**Affiliations:** ^1^ Department of Ecological and Biological Sciences University of Tuscia Viterbo Italy; ^2^ Department of Biological, Geological and Environmental Sciences University of Bologna Bologna Italy; ^3^ Institute of Research on Terrestrial Ecosystems National Research Council Porano (TR) Italy; ^4^ Human Spaceflight and Scientific Research Unit Italian Space Agency Rome Italy; ^5^ LE STUDIUM Institute for Advanced Studies Orléans France; ^6^ Institute of Polar Sciences National Research Council of Italy (CNR‐ISP) Messina Italy

## Abstract

The McMurdo Dry Valleys in Southern Victoria Land, Antarctica, are known for their extreme aridity, cold, and nutrient‐poor conditions. These valleys provide a valuable comparison to environments on Mars. The survival of microorganisms in these areas hinges on their ability to withstand dehydration due to the limited availability of liquid water. Some microorganisms have adapted to survive extended periods of metabolic inactivity and dehydration, a physiological response to the harsh conditions in which they exist. This adaptation is significant for astrobiology studies as it allows for testing the resilience of microorganisms under extraterrestrial conditions, exploring the boundaries and potential for life beyond Earth. In this study, we examined the survivability, metabolic activity, cellular membrane integrity, and ultrastructural damage of *Cryomyces antarcticus*, a eukaryotic organism used for astrobiological studies, following two dehydration processes. We conducted a fast dehydration process, simulating what happens on the surface of Antarctic rocks under typical environmental conditions, and a slow dehydration process, which is commonly used in astrobiological experiments. Our findings revealed a higher percentage of damaged cells following slow dehydration treatments, confirming that rapid dehydration reflects the adaptability of microorganisms to respond to sudden and drastic changes in the Antarctic environment.

## Graphical abstract

Active cells of the black fungus, *Cryomyces antarcticus*, were subjected to both long and short‐term dehydration processes. The responses of these fungal cells were evaluated using a variety of analyses, which included assessments of survivability, metabolic activity, cellular membrane integrity, and ultrastructural damage. It was observed that a higher percentage of cells were damaged in the long‐dehydrated samples. This finding underscores that short‐term dehydration better preserves the viability and integrity of *C. antarcticus* cells. This reflects the microorganism's adaptability in responding to rapid and drastic changes in the Antarctic environment.

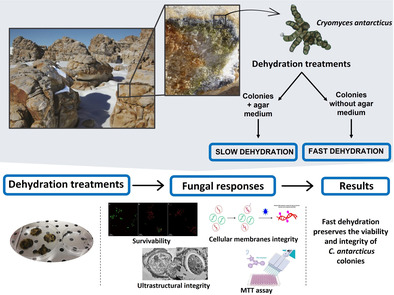

## INTRODUCTION

The ice‐free areas of McMurdo Dry Valleys (MDVs) in Antarctica represent one of the best terrestrial analogues for Mars environments due to their hyperarid, cold, and oligotrophic conditions associated with strong UV irradiation (Cassaro et al., 2021; Onofri et al., 2004; Wynn‐Williams & Edwards, 2000). The dryness is the main environmental characteristic of MDVs, enhanced by low availability of liquid water and high winds promoting water evaporation. The bioavailability of liquid water is the most important limiting factor for life and as a result, the survival of the Antarctic microorganisms in these areas is highly dependent on their resistance to dehydration. They have developed the ability to maintain metabolically inactive (dehydrated conditions) states, as a physiological adaptation to cope with the harsh environmental conditions in which they live. In fact, in these regions, the microorganisms are metabolically inactive for the majority of the year, except during the brief summer season when temperatures rise and water from snow or ice melt becomes available (Block et al., 2009; Friedmann, 1982; Selbmann et al., 2005; Sterflinger et al., 2012).

This strategy is also often exploited in astrobiology studies to test the resistance of different microorganisms under extraterrestrial conditions, to study the limits of life and the possibility of life elsewhere in the Universe, considering that hypothetical life forms might be present in a dormant form below the surface on other planets, such as Mars (Schulze‐Makuch et al., 2005). It has been widely demonstrated that several microorganisms have a higher chance of surviving real or simulated space conditions when exposed in a dehydrated condition than in a metabolically active state. This is even more relevant for irradiation treatment because the exposure of living systems to radiation could cause a direct ionization of DNA or ionization of water, resulting in the generation of radical species, which finally damages the living cells (Becker & Sevilla, 1993; von Sonntag, 2006).

The Antarctic black fungus *Cryomyces antarcticus*, extensively used as a eukaryotic test organism for astrobiological studies, has been reported to cope under different stressors, such as ionizing and non‐ionizing radiation, simulated space and Martian conditions, or real space conditions (Aureli et al., 2020; Cassaro et al., 2022; Onofri et al., 2015; Onofri et al., 2019; Pacelli et al., 2017; Pacelli et al., 2019; Pacelli, Cassaro, Aureli, et al., 2020). The majority of aforementioned studies were performed in its dehydrated state.

Although it has been reported that the fungus can cope with these stresses thanks to the dehydration condition in which it has been exposed, no studies focusing exclusively on the effects of dehydration have been conducted so far. In this context, we aimed to investigate the possible effects of dehydration itself on the fungus. In this preliminary work, we tested (i) a fast dehydration process, which simulates what may occur on Antarctic rock surfaces under typical environmental conditions (Gorbushina et al., 2008), and (ii) a slow dehydration process, which is commonly used in astrobiological experiments to dehydrate the fungus before exposure to various stressors. This approach enables a comprehensive twofold investigation into the impact of dehydration on the fungus and the validity of the method used in astrobiological research.

Our first hypothesis was that a longer dehydration process might create less damage in the fungus than a faster one since the cell would have more time to repair damage. To address this knowledge gap, fungal survivability, metabolic activity, cellular membrane integrity and ultrastructural damages were investigated.

## EXPERIMENTAL PROCEDURES

The test organism is the black fungus *Cryomyces antarcticus* CCFEE 515, isolated by R. Ocampo‐Friedmann from sandstone collected at Linnaeus Terrace in McMurdo Dry Valleys (Southern Victoria Land, Antarctica) by H. Vishniac, during the Antarctic expedition of 1980–1981 (Selbmann et al., 2005).

Fungal colonies were grown on Malt Extract Agar (MEA) medium (malt extract, powdered 30 g/L; agar 15 g/L; Applichem, GmbH) in Petri dishes and incubated at 15°C for 3 months.

Fast‐dehydrated fungal colonies were prepared by carefully removing colonies from the MEA cultivation medium using a sterile loop and placing them individually on the bottom of sterile Petri dishes. They were then left to undergo dehydration. Slowly dehydrated colonies were left to dehydrate within the Petri dishes along with the medium. To ensure complete dehydration, the weights of the two experimental sets (both Fast and Slow Dehydration) were determined at different time intervals using an analytical balance (refer to Tables S1 and S2). Each set of samples was weighed in its own Petri dish, both with and without the cultivation medium, under sterile conditions (the presence of the cultivation medium in slowly dehydrated samples explains the differences in the starting weights between the two experimental sets). All the following analyses were carried out on rehydrated Fast and Slow Dehydrated fungal colonies. Rehydration was performed immediately after the end of the dehydration process. Analyses were performed 72 h after rehydration, obtained by adding 1 mL of physiological water (0.9% NaCl). A number of untreated colonies were maintained hydrated (metabolically active) for the same time as the Fast and Slow Dehydrated colonies, respectively, and used as a control group. All experiments were carried out in triplicate.

To investigate the fungal colonies' survivability, the re‐hydrated colonies were treated with a LIVE/DEAD staining kit and visualized through a Laser Scanning Confocal Microscopy (Zeiss). The kit includes two nucleic acid stains that penetrate the cells: Propidium Iodide (PI) only penetrates cells with impaired cellular membranes, whereas SYTO 9 identifies all cells without distinguishing between those with intact and compromised membranes. This difference in staining allows for the distinction between living cells and dead ones.

The integrity of fungal cell membranes was investigated by Propidium MonoAzide (PMA) assay coupled with quantitative PCR (qPCR) according to the protocol reported in Pacelli et al. (2017). The PMA assay coupled with qPCR was used to discriminate damaged cells membrane to membrane‐intact cells. PMA is a compound that can covalently bind to DNA only in cells with a compromised cell membrane, thereby preventing PCR amplification.

Survivability and cellular membrane integrity results of Fast Dehydrated and Slow Dehydrated samples were expressed as a percentage compared with Control samples (100%, light blue bars).

To determine the metabolic activity of fungal colonies after the dehydration processes, the MTT (3‐(4,5‐dimethylthiazol‐2‐yl)‐2,5‐diphenyltetrazolium bromide) test was assessed. The assay is based on the ability of MTT dye, a yellow water‐soluble compound, to be reduced to purple‐coloured formazan crystals by mitochondrial succinate dehydrogenase only in metabolically active cells.

Lastly, the integrity of the ultrastructure was investigated using Electron Transmission Microscopy (TEM), as reported by Cassaro et al. (2022).

Detailed procedures are reported in the Data S1. Statistical analysis was performed using GraphPad Prism version 8 by comparing control and treated samples. Significant differences were calculated by Unpaired *t‐test* with *p < 0.05 and **p < 0.001.

## RESULTS

### Water loss

**FIGURE 1:**
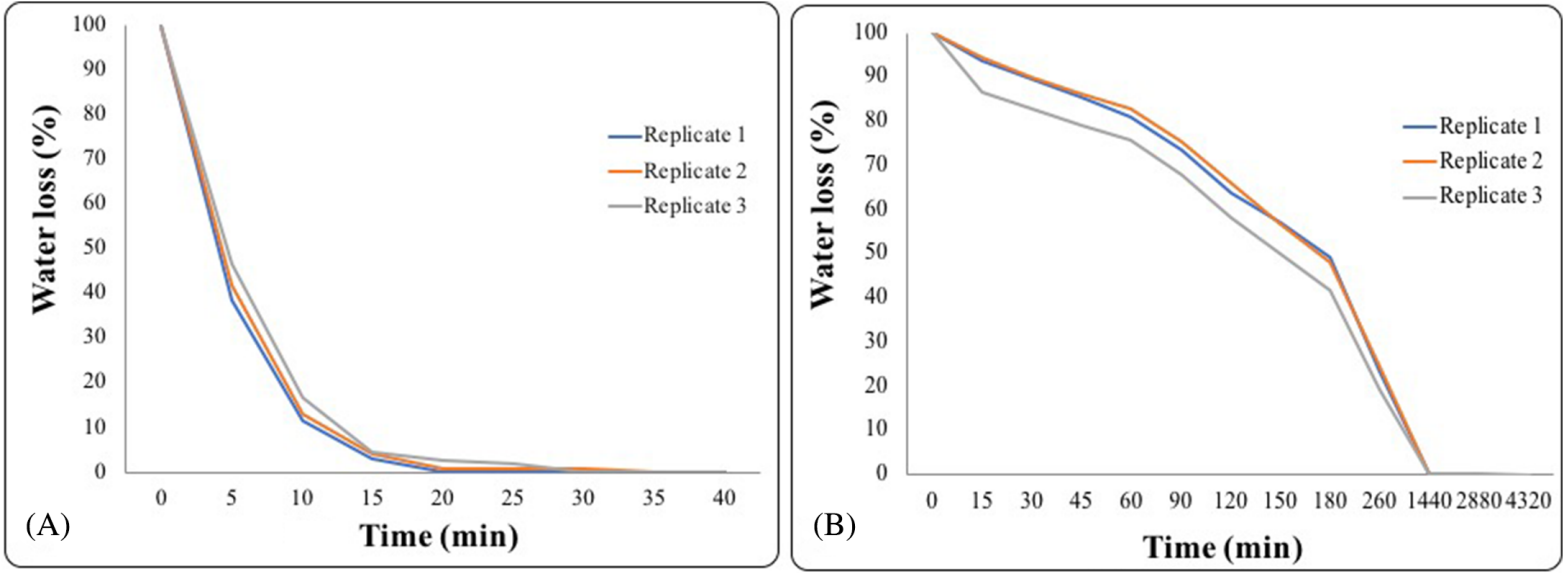
Water loss percentage with time (minutes) of *Cryomyces antarcticus* colonies after (A) fast and (B) slow dehydration process.

In colonies subjected to Fast Dehydration, constant weight (corresponding to the complete loss of free water) was reached in 40 min. In contrast, for colonies left to dry on MEA (Slow Dehydration), 72 h (4320 min) were necessary to achieve the constant weight (Figure 1A,B). All the results of water loss were reported in Tables S1 and S2.

### LIVE/DEAD staining and laser scanning confocal microscopy

**FIGURE 2:**
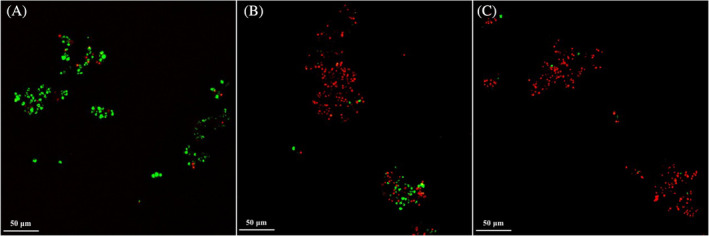
Confocal scanning laser microscopy images of fluorescent‐stained *Cryomyces antarcticus*. (A) Colonies grown under physiological conditions. (B) Colonies exposed to Fast dehydration. (C) Colonies exposed to Slow dehydration. Green fluorescence: cells with intact cellular membranes; red fluorescence: cells with damaged cellular membranes. (For interpretation of the references to colour in this figure legend, readers are directed to the LIVE/DEAD™ BacLight™ Bacterial Viability Kit, Catalogue number: L7007).

Laser scanning confocal microscopy images of *C. antarcticus* colonies stained with the LIVE/DEAD kit dyes after Fast and Slow Dehydration treatments are shown in Figure 2.

Overall, the highest number of colonies stained with red fluorescence, which was associated with damaged cellular membranes, was observed in Slow Dehydrated samples (82%), compared with Fast Dehydrated (62%) samples (Figures 2 and S1). Statistically significant differences in the survival of Fast and Slow dehydrated cells were reported (*p* < 0.05).

### Cellular membrane integrity assessment (PMA assay)

**FIGURE 3:**
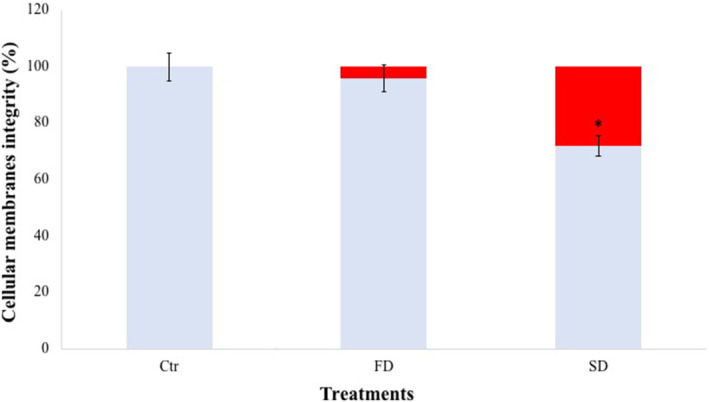
The cellular membrane integrity, measured with PMA assay coupled with qPCR of *Cryomyces antarcticus* colonies after FD and SD dehydration, expressed as a percentage compared with Control samples (100%, light blue bars). Undamaged cell‐membranes: light blue bars; damaged cell‐membranes: red bars. Ctr: *C. antarcticus* colonies grown under physiological conditions. FD, Fast dehydration; SD, Slow dehydration. Data were displayed as the mean ± percentage error bar. Significant differences between control (Ctr) and treated (FD and SD) samples were calculated byUnpaired *t* test with *p < 0.05.

Figure 3 shows the ratio between cells with damaged and undamaged cellular membranes in control samples and after Slow and Fast Dehydration treatments. Fast Dehydrated samples showed 4% damage to cellular membranes, whereas Slow Dehydrated samples showed a significantly higher proportion of damaged cell membranes (28%), compared to Control samples, which had 100% intact cellular membranes (Figure 3). Fast and Slow dehydrated cells showed statistically significant differences in cellular membrane integrity (*p* < 0.05).

### Assessment of metabolic activity through MTT assay

**FIGURE 4:**
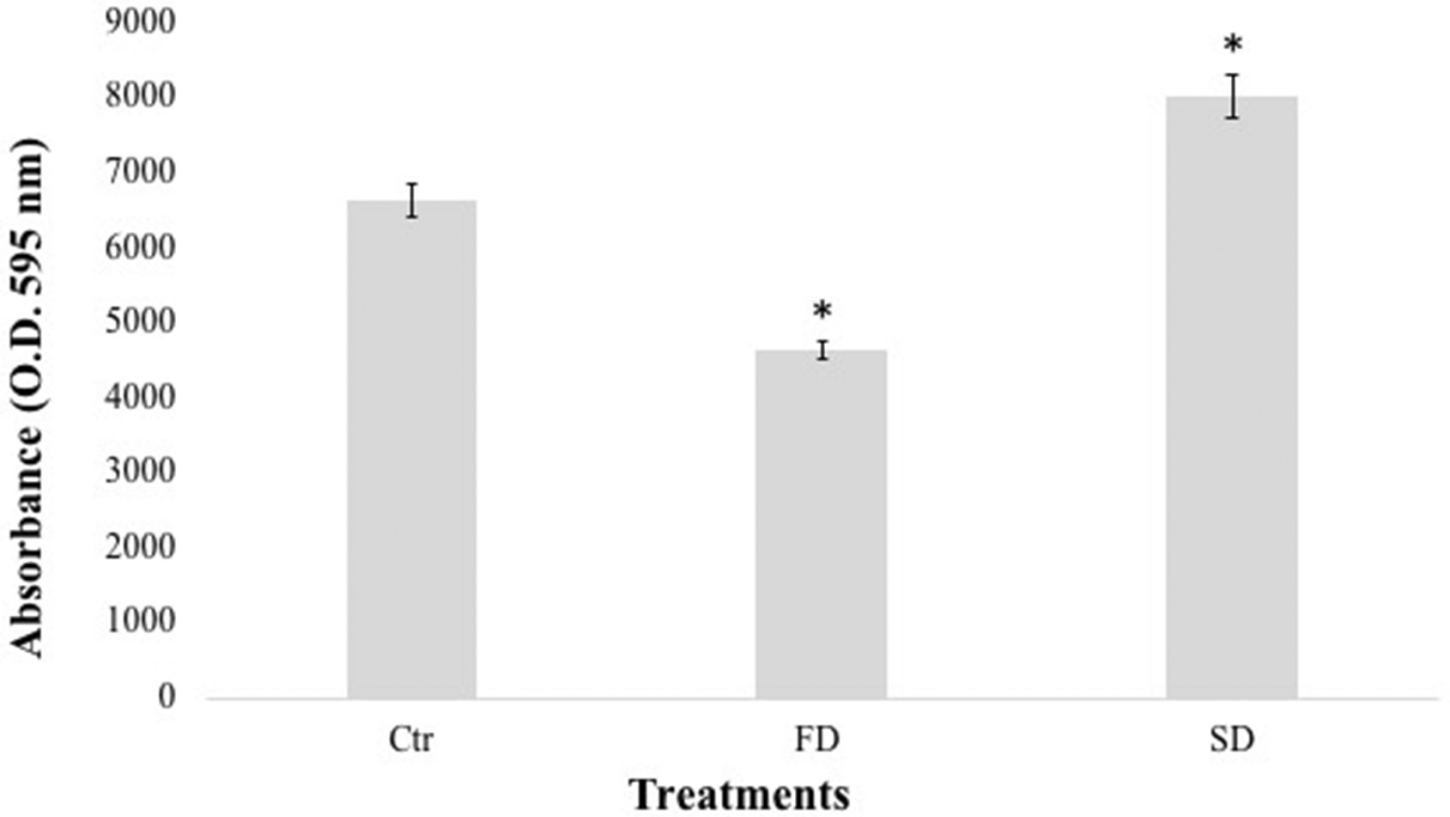
Effect of Fast (FD) and Slow (SD) dehydration treatments on the metabolic activity of *Cryomyces antarcticus* colonies, compred with Control (Ctr) samples. Showed results are after 72 h of incubation with the DMSO. Data were normalized against the control. Ctr: *C. antarcticus* colonies grown under physiological conditions. FD, Fast dehydration; SD, Slow dehydration. Data were displayed as the mean ± percentage error bar. Data were displayed as the mean ± percentage error bar. Significant differences between control (Ctr) and treated (FD and SD) samples were calculated by Unpaired *t* test with *p < 0.05.

In this study, the MTT assay was performed to detect differences in metabolic activity after Fast and Slow Dehydration treatments. During the MTT assay, the production of formazan was detected through spectrophotometric measurement, comparing control and dehydrated samples. The metabolic activity was tested after 48 and 72 h of DMSO addition; since no differences were reported among different incubation times, only the results after 72 h of incubation were reported (Figure 4). The results showed a significant increase in formazan production in Slow Dehydration samples, compared to Control and Fast Dehydration samples (Figure 4).

### Ultrastructural investigation: TEM observations

**FIGURE 5:**
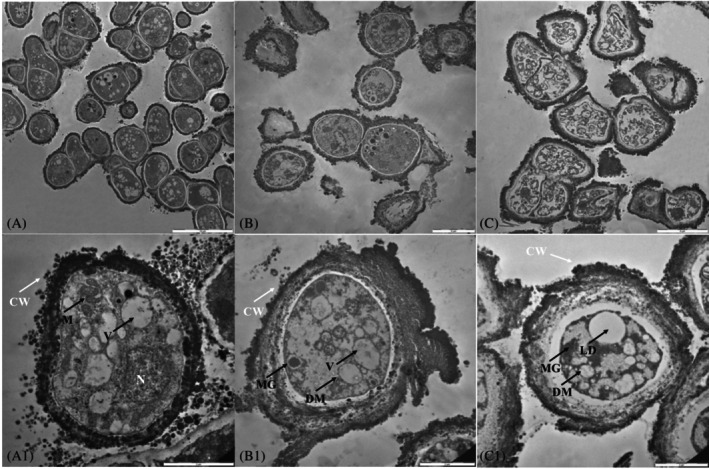
TEM micrographs overview of (A) Ctr colonies, (B) fast and (C) slow dehydration treatments of *Cryomyces antarcticus* cells. Scale bar: 5 μm. Details of TEM images of (A.1) untreated colonies (Ctr), (B.1) fast and (C.1) slow dehydration treatments of *C. antarcticus* cells. Scale bar: 2 μm. CW, cell wall; DM, damaged mitochondrium; LD, lipid droplet; M, mitochondrium; MG, melanin granules; N, nucleus; V, vacuoles.

TEM observations were performed on Control, Fast and Slow Dehydrated samples (Figure 5). Control samples showed well‐organized cells with dense and granular cytoplasm and well‐visible cellular structures. The nucleus, mitochondria and vacuoles were well preserved. Well‐preserved cellular membranes and cell walls have been reported (white arrows; Figure 5A,A.1). An overview of Fast Dehydration TEM micrograph showed the majority of good‐shape cells, even if nuclei and intracellular membranous structures were not always visible. Moreover, an increased number of vacuoles and melanin granules was reported (black and red arrows; Figure 5B,B.1). Differently, the Slow Dehydrated cells (Figure 5C) did not restore the cell shape after rehydration; nuclei and intracellular structures were not visible. The majority of cells had lost turgor, the cytoplasm was slack with an increased number of lipid inclusions (abundant coalescing lipid droplets) and melanin granules (black arrows; Figure 5C.1).

## DISCUSSION

One of the challenges to survive in the hyper‐arid environments is desiccation. In one of the driest and most Mars‐like environments on Earth, the McMurdo Dry Valleys, microcolonial black fungi have adapted to cope with this environmental stress by remaining dormant for most of the year (Sterflinger et al., 2012). This strategy is often adopted during astrobiological studies, where microorganisms are exposed to simulated or real space stressors in dehydrated conditions to prevent damage. Since dehydration tolerance relates to space stressor resistance, in this work we focused on the dehydration processes. We investigated the ability of the black fungus *Cryomyces antarcticus* to survive after fast and slow dehydration processes, occurring in 40 min and 72 h, respectively. Survivability, metabolic activity, cellular membrane integrity and ultrastructural damages of the fungus were investigated.

Although survival was obtained under all tested conditions, confocal microscopy image counts reported a higher death cell percentage in Slow Dehydrated compared to Fast Dehydrated samples (82%; Figure S1). Similarly, investigations on cell‐membrane integrity confirmed a higher percentage of cells with damaged cellular‐membranes in Slow Dehydrated (28%), compared to Fast Dehydrated (4%) and control samples (100%; Figure 3).

During Slow Dehydration treatment, colonies are forced to survive with ever lower levels of available water. A loss of cellular turgor and shape was reported for fungal cells. Indeed, cell volume significantly decreases, and the plasma membrane becomes tightly folded to maintain integrity (Rapoport, 2017).

Similarly, Gorbushina et al. (2008) reported an enhanced survival of the black microcolonial fungus *Sarcinomyces petricola* and an instant mycostasis after exposure to fast dehydration rather than following prolonged periods of low metabolic activity. In this work, colonies treated with a slow dehydration process drastically reduced the phospholipids content in association with the degradation of plasma membranes. These results were further confirmed by the decrease in red carotenoid levels in colonies exposed to a slow dehydration process, in comparison with colonies exposed to fast dehydration where levels remained unchanged and pigment synthesis was stimulated by the dehydration process (Gorbushina et al., 2008).

TEM images of slowly dehydrated cells of *C. antarcticus* showed a slack cytoplasm and an increase in lipid droplets. Similar results were previously obtained for *C. antarcticus* colonies grown in increasing concentrations of perchlorate solutions (Cassaro et al., 2022). Lipids located in lipid droplets have been reported as possibly associated with energy production via β‐oxidation, intracellular membrane synthesis, protein modification, signalling, secretion within lipoproteins, and cell reactions upon stress treatments (Barbosa et al., 2015; Klug & Daum, 2014). They may also be linked to the storage or inactivation of misfolded or aggregated proteins (Wang, 2016). These observations point to the possibility of lipid droplets actively participating in membrane structure repair and restoration processes that are activated during dehydration–rehydration processes.

In contrast, fast dehydration treatment, which mimics the environmental conditions of Antarctic rock surfaces, seemed to force the fungus to shift rapidly into a dormant state. In this case, TEM images reported a high number of cells in good shape, comparable with the cells in the control samples. These findings are consistent with those reported by Gorbushina et al. (2008), who found that the internal structures of the fast‐dehydrated cells of *S. petricola* were largely preserved, in contrast to slow dehydrated cells where lipid droplets were detected and intracellular membrane structures decayed.

TEM images also showed the presence of melanin granules. Fast dehydrated cells, in particular, showed sparse intracellular melanin granules, whereas in slow dehydrated cells, an increasing amount of melanin granules was reported. Melanin granules accumulate as a protection strategy against dehydration treatment, limiting cell‐wall permeability by reducing the size of cell‐wall pores (Jacobson & Ikeda, 2005). Also, melanin pigments may act as a constitutive antioxidant against dehydration (Cordero & Casadevall, 2017). However, these results differ from TEM observations of dehydrated cells of *S. petricola* (Gorbushina et al., 2008), where high levels of melanins and mycosporines were maintained as reserve storage compounds in fast dehydrated cells (Gorbushina et al., 2008). On the contrary, in slow dehydrated *S. petricola* colonies, small amounts of the protective pigments were detected, since new molecule production is avoided to minimize the metabolic cost (Gorbushina et al., 2008).

Finally, ultrastructural analysis of fast dehydrated cells showed the accumulation of vacuoles, which have a lot of functions including the regulation of cellular homeostasis. They may be critical for tolerating water stress, maintaining high cell turgor pressure, and enhancing water recall by lowering the potential water in cells (Cassaro et al., 2022; Pacelli, Cassaro, Maturilli, et al., 2020).

Despite the highest percentage of damaged cells in the Slow Dehydration treatment (39%), an increase in formazan production (metabolic activity) was reported compared to control and Fast Dehydration samples. This may be explained by an increase in mitochondrial mass, which can increase metabolic activity. As reported by Dupont et al. (2014) for *Saccaromyces cerevisiae*, over‐expression of Superoxide Dismutase (SOD) genes results in an eightfold increase in dehydration resistance. Mitochondrial SOD is involved in converting superoxide anions (O2 •–) into dioxygen and hydrogen peroxide and, therefore, essential for organism detoxification. In light of this, mitochondria appear to play a key role in dehydration processes. In our experiment, the potential activation of metabolic activity, suggested by the rise in formazan production, could be interpreted as a defensive response (e.g., mitochondrial respiration) against the treatment. Indeed, mitochondrial biogenesis is one of the main reactions in treated cells, as evidenced in numerous irradiation tests on *C. antarcticus*. This phenomenon leads to an increase in enzymatic MTT reduction (Pacelli et al., 2018; Pacelli et al., 2021; Rai et al., 2018).

Contrary to our original assumption, our findings suggest that rapid dehydration improves the viability and integrity of *C. antarcticus* colonies. These results reflect our test microorganism's high adaptability to drastic and rapid changes in the Antarctic environment on rock surfaces where freeze–thaw cycles are frequent. These findings suggest that *C. antarcticus* temporarily, but reversibly, suspends all metabolic processes as a result of the fast dehydration, maintaining a basal metabolism crucial for sustaining cell turgor. By secreting melanin granules, the fungus retains a small amount of water, increases osmolytes in the vacuoles and decreases pore walls. As previously reported by Zakharova et al. (2013), *C. antarcticus* responded to dehydration by down‐regulating its whole metabolism: any small additional protective proteins in response to dehydration were produced. These mechanisms are less energy‐consuming and might reflect the oligotrophic environment in which the fungus lives (Block et al., 2009).

This study is a first step toward understanding the relatively poorly known mechanism of the survival responses of the black fungus *C. antarcticus* following dehydration. Based on these early results, fast dehydrated samples have been chosen for advanced transcriptomic studies to investigate the processes underlying fungal resilience to rapid dehydration characterizing its natural environment.

## AUTHOR CONTRIBUTIONS

**Cassaro Alessia:** Conceptualization (equal); data curation (equal); formal analysis (equal); investigation (equal); methodology (equal); writing – original draft (equal). **D' Alò Federica:** Conceptualization (equal); data curation (equal); formal analysis (equal); investigation (equal); methodology (equal); writing – original draft (equal). **Pacelli Claudia:** Conceptualization (equal); resources (equal); supervision (supporting); writing – review and editing (supporting). **Onofri Silvano:** Funding acquisition (equal); project administration (equal); resources (equal); supervision (equal); validation (equal); writing – review and editing (equal). **Cavalazzi Barbara:** Supervision (equal); validation (equal); writing – review and editing (equal). **Zucconi Laura:** Supervision (equal); validation (equal); writing – review and editing (equal).

## CONFLICT OF INTEREST STATEMENT

The authors declare no conflict of interest.

## Supporting information


Data S1.

Figure S1.

## Data Availability

The data that support the findings of this study are available from the corresponding author upon reasonable request.
