# Supplementary material for: A preliminary survey of the cellular responses of the black fungus Cryomyces antarcticus to long and short‐term dehydration
Source: Environ Microbiol Rep. 2024 Jul 29;16(4):e13309. doi: 10.1111/1758-2229.13309 (PMC11286975; doi:10.1111/1758-2229.13309)
Supplement: Supplementary file 1 — Data S1. [file EMI4-16-e13309-s001.docx]

**A preliminary survey of the cellular responses of the** [**black fungus *Cryomyces antarcticus***](https://scholar.google.it/citations?view_op=view_citation&hl=it&user=FpTrP1UAAAAJ&citation_for_view=FpTrP1UAAAAJ:u5HHmVD_uO8C) **to long- and short-term dehydrations**

**Cassaro Alessia^1,2†^, D’Alò Federica^1,3†^, Pacelli Claudia^1,4*^, Cavalazzi Barbara^2,5^, Zucconi Laura^1,6^, Onofri Silvano^1^**

^1^Department of Ecological and Biological Sciences, University of Tuscia, Largo dell’Università snc, Viterbo, Italy

^2^Department of Biological, Geological and Environmental Sciences, University of Bologna, Via Zamboni 67, 40126, Bologna, Italy

^3^Institute of Research on Terrestrial Ecosystems, National Research Council, Viale Guglielmo Marconi, 2, 05010, Porano (TR), Italy

^4^Human Spaceflight and Scientific Research Unit, Italian Space Agency, via del Politecnico, Rome, Italy

^5^LE STUDIUM Institute for Advanced Studies, 45000 Orléans, France

^6^Institute of Polar Sciences, National Research Council of Italy (CNR-ISP), Spianata S. Raineri 86, 98122 Messina, Italy

^†^These authors have contributed equally to this work and share first authorship

**Running title:** Fungal cell responses to dehydration

**Supplementary materials**

**Materials and Methods**

**Determination of survivability through LIVE/DEAD Kit**

The fraction of live and dead fungal cells was assessed using a fluorescence assay immediately after the dehydration treatments, followed by sudden rehydration. The LIVE/DEAD BacLight Bacterial Viability Kit L13152 (Molecular Probes) was used to distinguish living cells from cells with compromised cell membranes. This kit includes two nucleic acid stains that differ in their ability to penetrate cells. SYTO 9 labels all cells in a population (with damaged and undamaged cellular membranes), while Propidium Iodide (PI) can only penetrate cells with damaged cellular membranes, allowing a reduction in SYTO 9 stain fluorescence. For these reasons we identify green fluorescing cells as live and red fluorescing cells as dead. For survival assay, the staining solution was prepared by mixing an equivalent volume of PI (18.3 mM) and SYTO 9 (1.67 mM), creating a solution. After the exposure to SD and FD treatments, cells were removed from the culture medium, washed twice with 300 μL PBS (1X) and centrifuged for 2 min at 14.000 rpm. The supernatant was removed and cells were resuspended into 300 μL PBS (1X). 3 μL of the solution were added to each sample and incubated for 15 min in dark conditions. After incubation, samples were centrifuged for 2 min at 14.000 rpm, the supernatant was removed and 100 μL PBS (1X) were used to resuspend the cells. For microscope observations, 5 μL of cell suspension were placed on a microscope slide and analyzed with a confocal microscope system (Zeiss LSM 710). Images were acquired using the interfaced software ZEN 2010: both microscope hardware and software configuration were always maintained. Technical parameters fixed in our acquisition procedure were pinhole size, at 1 AU (Airy unit), laser power and digital gain. Images were then processed using ImageJ software, in order to merge channels from monochrome acquisitions and make montage, when serial microscope scans of the specimen were performed along the z axis. Cells were counted using Image J software. After the cell count, the total number of colonies in each triplicate was calculated and the percentage of green and red cells to the total was determined.

**Membranes damage assessment** **Propidium MonoAzide (PMA) assay**

Quantitative PCR (qPCr) after PMA treatment was performed to assess membrane integrity after Fast and Slow Dehydration treatments. A solution of 5 μL of PMA (Biotium, Hayward, CA, United States) was added to treated micro-colonies and kept in the dark in a constant-shaking incubator for 1 h. PMA solution penetrates only damaged membrane cells and crosslinks the DNA preventing PCR after being exposed to light. DNA extraction, purification, normalization at the same concentration of 0.1 ng/mL was performed using the Qubit dsDNA HS Assay Kit (Thermo Fisher Scientific, Massachusetts, USA).  qPCR assay was performed to quantify the number of fungal Internal Transcribed Spacer (ITS) ribosomal DNA fragments (281 bp) present in treated and non-treated samples. A detailed protocol is provided by Onofri et al. (2012). All tests were performed in triplicate.

**Metabolic activity estimation by MTT (3-(4,5-dimethylthiazol-2-yl)-2,5-diphenyltetrazolium**

For the investigation of metabolic activity, a suspension containing 100 μL of fungal micro-colonies (3.5 x 10^5^ cells/mL) and a solution of 0.5 mg/mL of MTT in Phosphate-Buffered Saline (PBS) was placed into 96-well microplates. After incubation at room temperature for 24 and 72 hours, the MTT solution was removed with a multi-channel pipette, and 100 μL of DMSO (DiMethyl SulfOxide) was added. The absorbance of each treated well was read at 595 nm subtracting the absorbance relative to those wells only containing MTT. Absorption results were normalized by the number of cells per well, while these values were normalized by dividing the non-treated samples (Pos Ctr).

**Ultrastructural investigation: TEM observations**

Colonies exposed to SD and FD treatments were prepared for microscopy analyses according to the protocol reported in Pacelli et al. (2017). TEM observations were performed with a JEOL 1200 EX II electron microscope at the Great Equipment Center, section of Electron of the University of Tuscia (Viterbo, Italy). Micrographs were acquired with an Olympus SIS VELETA CCD camera and iTEM software.

**Figure legend**

**Figure S1.** Survival of *C. antarcticus* colonies after FD and SD treatments expressed as a percentage compared with Control samples. Numbers of survivors were determined following staining with the LIVE/DEAD Kit (see Fig. 1). Ctr: *C. antarcticus* colonies grown under physiological conditions. SD: Slow dehydration; FD: Fast dehydration. Light blue bars: alive cells; red bars: dead cells. Data were displayed as the mean ± percentage error bar. Significant differences were calculated by Unpaired *t test* with * = *p* < 0.05 and ** = *p* < 0.001. Significant differences between control (Ctr) and treated (FD and SD) samples were calculated by Unpaired *t test* with * = *p* < 0.05 and ** = *p* < 0.001.

**Tables**

**Table S1.** Water loss values of *C. antarcticus* colonies exposed to a fast dehydration process

|  | | **Tray** | **Starting time** | **5 min** | **10 min** | **15 min** | **20 min** | **25 min** | **30 min** | **35 min** | **40 min** |
| --- | --- | --- | --- | --- | --- | --- | --- | --- | --- | --- | --- |
|  |  |  | **(±SD)** | **(±SD)** | **(±SD)** | **(±SD)** | **(±SD)** | **(±SD)** | **(±SD)** | **(±SD)** | **(±SD)** |
| **Colonies + Tray (*)** | 5.9764  (±0.0006) | | 5.9940  (±0.0037) | 5.9869  (±0.0026) | 5.9834  (±0.0017) | 5.9821  (±0.0012) | 5.9818  (±0.0012) | 5.9818  (±0.0011) | 5.9817  (±0.0010) | 5.9816  (±0.0010) | 5.9816  (±0.0010) |
|  |  | |  |  |  |  |  |  |  |  |  |
| **Colonies - Tray (*)** |  | | 0.0175  (±0.0035) | 0.0105  (±0.0024) | 0.0069  (±0.0015) | 0.0057  (±0.0010) | 0.0054  (±0.0010) | 0.0053  (±0.0009) | 0.0052  (±0.0008) | 0.0052  (±0.0008) | 0.0052  (±0.0008) |
|  |  | |  |  |  |  |  |  |  |  |  |
| **g H_2_O** |  | | 0.0123  (±0.0000) | 0.0053  (±0.0017) | 0.0017  (±0.0007) | 0.0005  (±0.0002) | 0.0002  (±0.0002) | 0.0001  (±0.0002) | 0.0000  (±0.0001) | 0.0000  (±0.0000) | 0.0000  (±0.0000) |
|  |  | |  |  |  |  |  |  |  |  |  |
| **% H_2_O** |  | | 100  (±0.0000) | 42.3813  (±4.0808) | 13.6761  (±2.6888) | 3.9413  (±0.7748) | 1.1577  (±1.3676) | 0.9355  (±1.0062) | 0.2688  (±0.4656) | 0  (±0.0000) | 0  (±0.0000) |
|  |  | |  |  |  |  |  |  |  |  |  |
| **% Water loss** |  | | 0  (±0.0000) | 57.6187  (±4.0808) | 86.3239  (±2.6888) | 96.0587  (±0.7748) | 98.8423  (±1.3676) | 99.0645  (±1.0062) | 99.7312  (±0.4656) | 100  (±0.0000) | 100  (±0.0000) |
|  |  | |  |  |  |  |  |  |  |  |  |
| **Starting weight** | 0.0175  (±0.0035) | |  |  |  |  |  |  |  |  |  |
|  |  | |  |  |  |  |  |  |  |  |  |

(*) All the values are expressed in g.

**Table S2.** Water loss values of *C. antarcticus* colonies exposed to slow dehydration process (*) All the values are expressed in g.

|  | **Tray** | **Starting time**  **(±SD)** | **15 min**  **(±SD)** | **30 min**  **(±SD)** | **45 min**  **(±SD)** | **1 h**  **(±SD)** | **1 h 30 min**  **(±SD)** | **2 hours**  **(±SD)** | **2 h 50**  **min**  **(±SD)** | **3 h**  **(±SD)** | **4 h 20 min**  **(±SD)** | **24 h**  **(±SD)** | **48 h**  **(±SD)** | **72 h**  **(±SD)** |
| --- | --- | --- | --- | --- | --- | --- | --- | --- | --- | --- | --- | --- | --- | --- |
| **Colonies + Tray (*)** | 5.9763  (±0.0008) | 7.5744  (±0.0661) | 7.4859 (±0.0675) | 7.4243 (±0.0638) | 7.3677 (±0.0638) | 7.3072 (±0.0675) | 7.1936  (±0.0678) | 7.0468 (±0.0678) | 6.9061  (±0.0665) | 6.7725  (±0.0667) | 6.4188  (±0.0558) | 6.0968  (±0.0099) | 6.0955  (±0.0097) | 6.0954  (±0.0097) |
| **Colonies -**  **Tray (*)** |  | 1.5980  (±0.0669) | 1.5095  (±0.0684) | 1.4479  (±0.0636) | 1.3913  (0.0641) | 1.3308  (±0.0683) | 1.2172  (±0.0677) | 1.0705  (±0.0676) | 0.9297  (±0.0670) | 0.7961  (0.0666) | 0.4425  (0.0556) | 0.1205  (0.0099) | 0.1186  (±0.0098) | 0.1185  (±0.0098) |
| **g H_2_O** |  | 1.5202  (±0.0715) | 1.3910  (±0.0609) | 1.3294  (±0.0559) | 1.2728  (±0.056) | 1.2123  (±0.0601) | 1.0987  (±0.0602) | 0.9520  (±0.0605) | 0.8112  (±0.0593) | 0.6776  (±0.0584) | 0.3239  (±0.0478) | 0.0015  (±7,0711E-05) | 0.0001  (±0.0001) | 0 |
| **% H_2_O** |  | 100  (±0.0000) | 91.59  (±4.4423) | 87.53  (±4.0439) | 83.80  (±3.905) | 79.80  (±3.6444) | 72.33  (±3.9323) | 62.67  (±3.9412) | 54.55  (±4.1147) | 46.26  (±3.9651) | 22.42  (±2,8138) | 0.10  (±0.0057) | 0.01  (±0.0065) | 0 |
| **% Water**  **loss** |  |  | 8.4126  (±4.4424) | 12.4709  (±4.0439) | 16.2038  (±3.905) | 20.2027  (±3.6446) | 27.6793  (±3.9323) | 37.3391  (±3.9412) | 45.4504  (±4.1147) | 53.7478  (±3.9651) | 77.5898  (±2.8138) | 99.9006  (±0.0057) | 99.9937  (±0.0054) | 100  (±0.0000) |
| **Starting**  **weight** | 1.5980  (±0.0008) |  |  |  |  |  |  |  |  |  |  |  |  |  |

(*) All the values are expressed in g.
