# Supplementary figures and images for: A preliminary survey of the cellular responses of the black fungus Cryomyces antarcticus to long and short‐term dehydration
Source: Environ Microbiol Rep. 2024 Jul 29;16(4):e13309. doi: 10.1111/1758-2229.13309 (PMC11286975; doi:10.1111/1758-2229.13309)

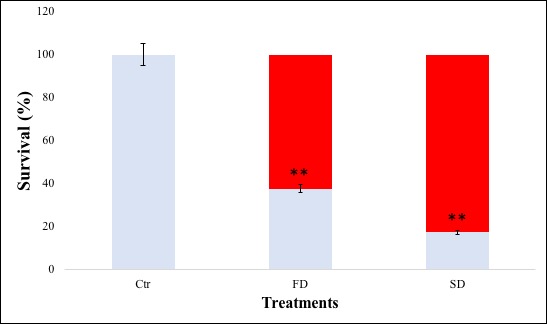

Supplement: Supplementary file 2 — Figure S1. [file EMI4-16-e13309-s002.jpg]
